# Expression and activation of the oxytocin receptor in airway smooth muscle cells: Regulation by TNFα and IL-13

**DOI:** 10.1186/1465-9921-11-104

**Published:** 2010-07-29

**Authors:** Yassine Amrani, Farhat Syed, Chris Huang, Katherine Li, Veronica Liu, Deepika Jain, Stefan Keslacy, Michael W Sims, Hasna Baidouri, Philip R Cooper, Hengjiang Zhao, Salman Siddiqui, Christopher E Brightling, Don Griswold, Lily Li, Reynold A Panettieri

**Affiliations:** 1 Centocor Research & Development Inc., 200 Great Valley Parkway, Malvern, PA 19355, USA; 2 Pulmonary, Allergy and Critical Care Division, Department of Medicine, University of Pennsylvania, TRL Suite 1200, 125 South 31st Street, Philadelphia, PA 19104, USA; 3 Therakos, 437 Creamery Way, Exton, PA 19341. USA; 4 Institute for Lung Health and Department of Infection, Immunity and Inflammation, University of Leicester, UK

## Abstract

**Background:**

During pregnancy asthma may remain stable, improve or worsen. The factors underlying the deleterious effect of pregnancy on asthma remain unknown. Oxytocin is a neurohypophyseal protein that regulates a number of central and peripheral responses such as uterine contractions and milk ejection. Additional evidence suggests that oxytocin regulates inflammatory processes in other tissues given the ubiquitous expression of the oxytocin receptor. The purpose of this study was to define the role of oxytocin in modulating human airway smooth muscle (HASMCs) function in the presence and absence of IL-13 and TNFα, cytokines known to be important in asthma.

**Method:**

Expression of oxytocin receptor in cultured HASMCs was performed by real time PCR and flow cytomery assays. Responses to oxytocin was assessed by fluorimetry to detect calcium signals while isolated tracheal rings and precision cut lung slices (PCLS) were used to measure contractile responses. Finally, ELISA was used to compare oxytocin levels in the bronchoalveloar lavage (BAL) samples from healthy subjects and those with asthma.

**Results:**

PCR analysis demonstrates that OXTR is expressed in HASMCs under basal conditions and that both interleukin (IL)-13 and tumor necrosis factor (TNFα) stimulate a time-dependent increase in OXTR expression at 6 and 18 hr. Additionally, oxytocin increases cytosolic calcium levels in fura-2-loaded HASMCs that were enhanced in cells treated for 24 hr with IL-13. Interestingly, TNFα had little effect on oxytocin-induced calcium response despite increasing receptor expression. Using isolated murine tracheal rings and PCLS, oxytocin also promoted force generation and airway narrowing. Further, oxytocin levels are detectable in bronchoalveolar lavage (BAL) fluid derived from healthy subjects as well as from those with asthma.

**Conclusion:**

Taken together, we show that cytokines modulate the expression of functional oxytocin receptors in HASMCs suggesting a potential role for inflammation-induced changes in oxytocin receptor signaling in the regulation of airway hyper-responsiveness in asthma.

## Introduction

Oxytocin, a hypothalamic neuropeptide, induces uterine contractions during parturition and milk ejection during lactation via activation of the oxytocin receptor, a G protein-coupled receptor [1]. Prior to the onset of labor, uterine muscle becomes exquisitely sensitive to oxytocin due to dramatic increases in the expression of oxytocin receptors (OXTR) [2] whose activation promotes myometrial shortening. Recently, the role of oxytocin has expanded given the discovery of OXTR gene expression in diverse tissues such as the pituitary, kidney, ovary, testis, thymus, heart, vascular endothelium, osteoclasts, myoblasts, pancreatic islets, adipocytes, several types of cancer cells [1], smooth muscle and epithelial compartment of the human epididymis [3]. Evidence also suggests oxytocin may serve as an acute phase response protein after injury [1]. Whether oxytocin plays a role in lung diseases remains unknown.

Asthma, a chronic disorder with a genetic and an environmental component [4], manifests as reversible airway obstruction, airway hyper-responsiveness, inflammation and remodeling. Asthma affects an estimated 15 million Americans, and asthma morbidity and mortality continues to rise globally [5]. Airway inflammation associated with the mucosal infiltration of T helper (Th)2 subset of CD4^+ ^T cells and eosinophils [6,7] evokes the production of various pro-inflammatory mediators involved in the pathogenesis of asthma [5]. Evidence suggests that the incidence of asthma exacerbations during pregnancy occurs in about 20% of women [8,9]. Why asthma may worsen during pregnancy in some women remains unclear. The role of gonadal hormones in altering lung function during pregnancy has been proposed as a possible explanation for asthma morbidity in pregnancy [10,11]. A direct action of estrogen on airway smooth muscle function has been recently reported as a plausible molecular mechanism linking sex hormone and disease worsening [12]. Other circulating pregnancy-associated factors also exert detrimental effects on asthma by modulating airway smooth muscle function. Evidence suggests that oxytocin can act as a potent immune-modulary factor in animal models of myocardial infarction [13], atherosclerosis [14] or pyelonephritis [15]. Although the oxytocin receptor is expressed in the lungs [16], whether oxytocin plays a role in airway diseases remains unknown.

Of the resident airway tissues, airway smooth muscle, a target and/or producer of inflammatory cytokines, represents the pivotal tissue regulating bronchomotor tone [17,18]. We postulate that oxytocin modulates airway smooth muscle shortening and that pro-inflammatory cytokines, interleukin (IL)-13 and tumor necrosis factor TNFα, may amplify oxytocin's effects on airway smooth muscle [19]. Previous studies demonstrate that calcium mobilization induced by oxytocin in human myometrial cells was enhanced with TNFα treatment [20]. This is an interesting finding since we have also shown that TNFα amplifies calcium responses evoked by other agonists including carbachol, bradykinin and histamine [21,22]. Others, using murine models of allergen-induced inflammation, have implicated TNFα and IL-13 in promoting airway hyper-responsiveness inflammation and mucus hyper-secretion [23-27].

Here, we report the expression and function of oxytocin receptor in airway smooth muscle (both in human and mouse); more importantly, we also report that oxytocin receptor expression is affected by IL-13 and TNFα. In addition, measurable oxytocin levels are found in BAL fluid in healthy subjects and those with asthma. Although the clinical relevance of these findings remains to be determined, the ability of cytokines to evoke oxytocin responses raises a novel hypothesis that expression or coupling of oxytocin receptors, not oxytocin levels per se, in the airways promotes airway hyperresponsiveness.

## Materials and methods

### Subjects

Subjects with asthma and healthy controls were recruited from Glenfield Hospital outpatients, staff, and by local advertising. Asthma was defined by one or more of the following objective criteria: significant bronchodilator reversibility of FEV_1 _> 200 ml, a provocation concentration of methacholine causing a 20% fall in FEV_1 _(PC_20_) of less than 8 mg/ml or a peak flow amplitude % mean over 2 weeks of more than 20%. Asthma severity was classified using the current GINA guidelines based upon the GINA treatment steps (GINA guideline). Normal subjects had no history of respiratory disease and normal spirometry and methacholine responsiveness. All subjects were non-smokers with a past smoking history of less than 10 pack years. The Leicestershire Ethics Committee approved the study and all patients gave their written informed consent.

### Protocol and clinical measurements

Subjects attended on two occasions. At the first visit, spirometric parameters before and after bronchodilator (400 μg inhaled albuterol) and methacholine airway responsiveness using the tidal breathing method (0.03 to 16 mg/ml) were determined. At the second visit 1 week later, the subjects underwent bronchoscopy and a 180 ml BAL into the middle lobe. The BAL was centrifuged and the cell-free supernatant stored at - 80°C for later analysis.

### Oxytocin assay

Oxytocin levels in BAL samples from asthmatic and healthy subjects were assayed using a competitive enzyme immunoassay kit (ELISA) from Assay Designs, Inc. catalog no. 901-153 (Ann Arbor, MI, USA). Assay procedure and calculation of the concentration of oxytocin were performed using the protocol provided by the kit. Briefly, 100 μl of all standards and BAL samples were loaded in triplicate with 50 μl of blue conjugate antibody into each well, except the total activity and blank wells. The plate was sealed and incubated at 4°C for 24 hr. After incubation, wells were washed 3 times, and pNpp substrate solution was added and incubated at room temperature for 1 hr. Subsequently, the plates were read immediately at an optical density of 405 nm. The concentration of oxytocin in the samples was calculated based on the standard curve obtained with known concentrations of oxytocin provided in the kit.

### Human airway smooth muscle cell culture

Human tracheae were obtained from lung transplant donors, in accordance with procedures approved by the University of Pennsylvania Committee on Studies Involving Human Beings. A segment of trachea just proximal to the carina was removed under sterile conditions and the tracheal muscle was isolated. Enzymatic dissociation of the tissue was performed for 90 min in a shaking water bath at 37°C. The cell suspension was filtered through 105 μm Nytex mesh, and the filtrate was washed with equal volumes of cold Ham's F12 medium (Gibco BRL Life Technologies, Grand Island, NY) supplemented with 10% FBS (HyClone, Logan, UT), 100 U/ml penicillin (Gibco), 0.1 mg/ml streptomycin (Gibco), and 2.5 μg/ml fungizone (Gibco). Aliquots of the cell suspension were plated at a density of 1 × 104 cells/cm2. The cells were cultured in Ham's F12 media supplemented with 10% FBS, 100 U/ml penicillin, 0.1 mg/ml streptomycin and this was replaced every 72 hr. Human ASM cells in subculture during the second through fifth cell passages were used. Primary human airway smooth muscle cells from "normal" donors were cultured with IL-13 or a naturally occurring mutant form IL13R130Q (50 ng/ml) or TNFα (10 ng/ml) for 0, 6 or 18 hr after resting the cells for 24 hr.

### Reverse transcription and real time PCR

Real time PCR was performed to assess whether oxytocin receptor expression was modulated by pro-inflammatory cytokines, TNFα, IL- 13 and IL-13R130Q, a naturally occurring isoform of IL-13 and associated with high serum IgE levels [28]. Total RNA was isolated using the RNeasy mini kit (Qiagen, Inc., Valencia, CA) as per manufacturer's instructions. 1 μg of total RNA was reverse transcribed as per protocol using TaqMan^® ^RT reagents (Applied Biosystems) at 37°C for 120 min followed by 25°C for 10 min. Forty ng of cDNA per reaction was used in the real time PCR using the ABI Prism^® ^7900 HT Sequence Detection System (Foster City, CA). In the presence of AmpliTaq Gold DNA polymerase (ABI Biosystems, Foster City, CA), the reaction was incubated for 2 min at 50°C followed by 10 min at 95°C. Then the reaction was run for 40 cycles at 15 sec, 95°C and 1 min, 60°C per cycle. Assays-on-Demand™ primers and probes specific for oxytocin receptor (Applied Biosystems; ID number Hs00168573_m1) were used in the PCR. The endogenous 18 S rRNA was measured and used to normalize all samples using the ΔΔCT method (Applied Biosystems). Gene expression level of OXTR is expressed relative to 18 S and untreated samples in each stimulation study, respectively. At least 3 (often 6) replicates were run for each condition. Student T-test was conducted between a pair of treatment conditions to determine if the observed difference in expression value was statistically significant, use p < 0.05 as threshold.

### Flow cytometry

Flow cytometry was performed to determine whether cytokine-induced changes in receptor mRNA expression is associated with changes in protein expression as described previously with slight modifications [29]. Briefly, adherent cells treated with 50 ng/nl IL-13 or 10 ng/ml TNFα for 24 hr were washed with PBS, detached by trypsinization (2 min, 37°C) and then washed with Ham's-F12 (10% FCS) media, centrifuged, and transferred to microfuge tubes (1.5 ml). Cells were resuspended in fixation medium A and then permeabilization medium B (Caltag, Burlingame, CA). Cells were then incubated with either control isotype of goat polyclonal anti-human oxytocin receptor antibody (2 μg/ml, Santa Cruz Biotech., CA), washed, and followed by 1 hr incubation with a fluorescein isothiocyanate-conjugated secondary antibody (1:100, Jackson ImmunoResearch Laboratories, West Grove, PA). The cells were then centrifuged and resuspended in cold PBS in microfuge tubes. Samples were then analyzed using an EPICS XL flow cytometer (Coulter, Hialeah, FL). Levels of oxytocin receptor were expressed as the percent increase in mean fluorescence intensity over unstimulated cells. Oxytocin levels in the sputum supernatant of asthmatics and healthy control subjects were compared using the Wilcoxon's ranksum test. Multivariable linear regression was used to determine whether oxytocin levels differed between the two groups after correcting for potential confounders. Age, sex, percent predicted forced expiratory volume in one second, and inhaled corticosteroid dose were added into the model with disease state one at a time to determine if they altered the effect of disease on oxytocin levels.

### Cytosolic free calcium

To further determine whether changes in receptor expression were associated with increased receptor function, we first tested whether oxytocin triggers calcium responses in human ASM cells and whether these responses were affected following treatment with cytokines. Calcium measurements were performed as described previously [30]. Briefly, growth-arrested HASM cells stimulated with 50 ng/ml IL-13 for 24 hr were loaded with 3 μM Fura-2/AM in HEPES buffer and resuspended (at 10^6 ^cells/ml) in 1-cm quartz cuvettes. After preincubating at 37°C for 2 min with gentle stirring, changes in Fura fluorescence were measured with a PTI fluorimeter (Photon Technology International, Inc.) after addition of oxytocin (0.1-1 mM). Thrombin or bradykinin was used as positive controls as these agonists have previously shown to increase calcium mobilization in HASM cells [31].

### Measurement of contractile responses

Whether oxytocin induces contractile responses in airway smooth muscle has not been tested. Based on the oxytocin ability to trigger calcium responses in cultured ASM cells, we next investigate whether oxytocin also stimulates contractile responses using two different experimental approaches:

#### (1) Isometric force generation

As described in our previous reports [32-35], tracheal smooth muscle reactivity was analyzed using temperature-controlled (37°C) myographs (Organ Bath Model 700MO, J.P. Trading, Aarhus, Denmark) containing 5 ml of Krebs-Henseleit (K-H) (118 NaCl, 4.7 KCl, 1.2 KH_2_PO_4_, 11.1 dextrose, 1.2 MgSO_4_, 2.8 CaCl_2 _and 25 NaHCO_3_) that was continuously aerated with a 5% CO_2 _and 95% O_2 _mixture; a pH of 7.40-7.45 was established for the entire duration of the experiments. The tracheal segments were mounted on two L-shaped metal pins. One pin was connected to a force-displacement transducer for continuous recording of isometric tension by the Chart software (AD Instruments Ltd., Hastings, UK). The other pin is directly connected to a displacement device, allowing the adjustment of the basal tensions that were set at approximately 0.5 g and stimulated with agonists after attainment of steady-state tension. All values were expressed as means ± SE. Student's unpaired t-test was used to compare the effect of oxytocin or carbachol. A P value of < 0.05 was considered significant.

#### (2) Precision cut lung slices (PCLS)

PCLS was performed as described previously [36,37]. Female Balb C mice (8-10 weeks) were euthanized by an overdose to carbon dioxide gas. The trachea was exposed and a cannula was inserted. Lungs were inflated using 1.0 ml of a 2% (w/v) low melting point agarose solution followed by a 0.1 ml air bolus to force the agarose out of the airways and into the alveoli. The inflated lungs were dissected from the thoracic cavity and mounted in agarose using the tissue embedding kit (Alabama Research & Development, Model # MD2200). Cores were placed into the slicer (Krumdieck Tissue Slicer, Alabama Research & Development, Model # MD4000) and the speed was set to produce slices at approximately 1 per 30 seconds (thickness: 250 μm). Suitable airways on slices were selected on the basis of the following criteria: presence of a full smooth muscle wall (i.e., cut perpendicular to direction of airway), presence of beating cilia to eliminate blood vessels, and unshared muscle walls at airway branch points to eliminate possible counteracting contractile forces. Slices were then transferred to incubating buffer and incubated at 37°C on a rotating platform. Trauma caused by tissue slicing contracts the airway presumably by the release of mediators. The incubation buffer was therefore changed every 30 min for 4 hr to remove any constrictor mediators released from the tissue that prevents the airway from relaxing to baseline, and then every 24 hr as indicated. On the next day (18 hr later), slices were washed again with fresh medium.

Lung slices were then placed in a 12-well plate in 1.0 ml assay buffer. The airway was located using a microscope (Nikon ECLIPSE, Model # TE2000-U; Mag.: x100) and the slice was held in place using a platinum weight with nylon attachments. Media was added for baseline measurements. The airway was positioned so a live video feed (Evolution QEi; Model # 32- 0074A-130 video-recorder) could be viewed. A baseline image was taken followed by the administration of the 1.0 μM concentration of oxytocin. Images were collected after 4 min or after no further contraction. The airways were then administered a 1.0 μM concentration of carbachol followed by the collection of images.

Airway lumen size was measured using a macro written within Image Pro-Plus (version 6.0) software (Media Cybernetics) and given in units of μm2. After functional studies, the area of each airway at baseline and at the end of each dose of agonist was calculated using the same macro written within Image Pro-Plus software. Data were plotted as percentage contractions (100 - (% Initial Airway Size)) for each agonist. Data were expressed as means ± SEM. Statistical difference was shown by using a paired *t *test.

## Results

### Expression of total mRNA for the oxytocin receptor

**Figure 1:**
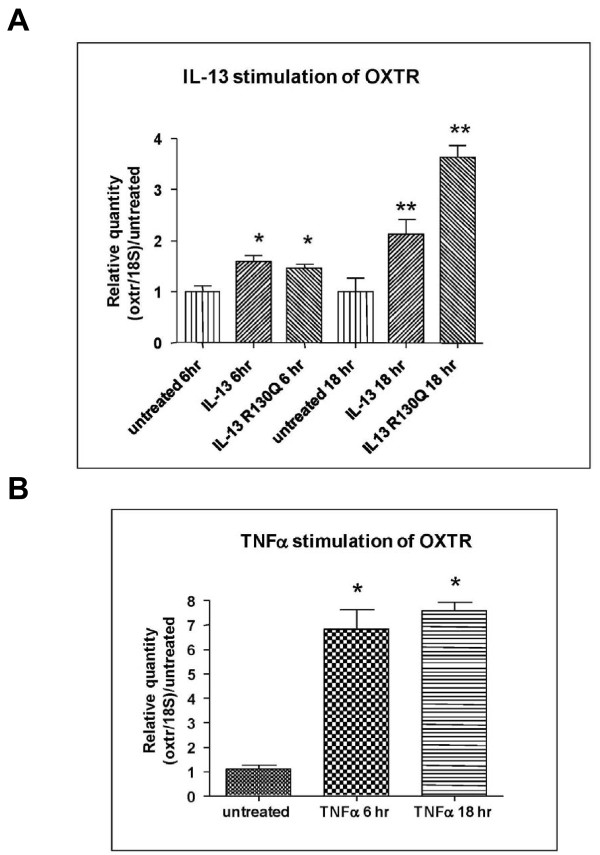
**Real time PCR (TaqMan^®^) analysis showing the quantity of oxytocin receptor normalized to 18 S and relative to untreated in primary human airway smooth muscle cells from normal donors when treated with IL-13 or IL-13R130Q (A) and TNFα (B) for 6 hr and 18 hr**. * Significant difference between treated and untreated conditions (p < 0.05), but not between the two treated conditions; **Significant difference between treated and untreated conditions (p < 0.05), as well as between the two treated conditions.

Primary human airway smooth muscle monolayers from healthy donors were cultured with IL-13 or a naturally occurring mutant form IL-13R130Q for 0, 6 hr or 18 hr at 50 ng/ml after serum deprivation for 24 hr. Total RNA was isolated, reverse transcribed and used in real time PCR analysis for oxytocin receptor which revealed that oxytocin receptor was expressed in primary airway smooth muscle cells, and the expression was increased by IL-13 (2 fold) and by IL-13R130Q (2 fold) at 6 hr and approximately 3.5 fold at 18 hr (Figure 1A). In parallel, airway smooth muscle cells were also treated with tumor necrosis factor alpha (TNFα) for 0, 6 hr or 18 hr at 10 ng/ml. Total mRNA expression for oxytocin receptor showed that TNFα increased expression of oxytocin receptor by approximately 5 fold at 6 hr and 7 fold at 18 hr as compared with that obtained from diluent-treated cells (Figure 1B).

To characterize the expression of oxytocin receptor across various tissues, real time PCR was performed to detect oxytocin receptor in normal tissues. The highest expression was detected in breast, followed by male caval vein and penis and uterus. The basal expression level was modest for the majority of normal tissues, including normal lung. These data suggest that OXTR expression in lung occurs at low levels in the basal state (Table 1).

**Table 1:** The expression of oxytocin receptor across various tissues as measured by real time PCR. The expression is normalized to 18 S.

Total RNA Human	18 S Qty adjusted	Total RNA Human	18 S Qty adjusted
Adrenal, Female, Adult	1	Kidney, Male, Adult	2.7
Aorta, Female, Fetal	3.1	Kidney, Male, Fetal	4.4
Bladder, Male, Adult	3.4	Larynx, Male, Adult	5.8
Bladder, Female, Fetal	5.4	Larynx, Male, Adult	3.3
Bladder, Male, Fetal	7.2	Liver, Female, Adult	0.44
Brain, Female, Fetal	10.3	Liver, Female, Fetal	1.3
Brain, Male, Adult	9.0	Liver, Male, Adult	0.6
Brain, Male, Fetal	2.6	Liver, Male, Fetal	2
Brain, Occipital Cortex, Male, Adult	11.6	**Lung, Female, Adult**	**1.9**
Brain, Parietal Cortex, Male, Adult	8.3	**Lung, Female, Fetal**	**1.8**
**Breast, Female, Adult**	**160.2**	Lung, Male, Adult	3.5
Caval Vein, Male, Adult	25.3	Lung, Male, Fetal	8.3
Cervix, Female, Adult	1.7	Lymph Node, Male, Adult	6.6
Colon, Ascending, Female, Adult	12.4	Ovary, Female, Adult	11.5
Colon, Descending, Female, Adult	1	Pancreas, Male, Adult	0.28
Colon, Female, Fetal	2.6	Parotid, Female, Adult	0.41
Colon, Male, Adult	3.2	Penis, Male, Adult	25.3
Colon, Male, Fetal	1.9	Pericardium, Male, Adult	2.7
Heart, Female, Adult	0.16	Placenta, Adult, Female	6
Heart, Female, Fetal	12	Prostate, Male, Adult	4.2
Heart, Left Atrium, Male, Adult	0.86	Rectum, Male, Adult	0.78
Heart, Male, Adult	0.33	Skeletal Muscle, Female, Fetal	1.8
Kidney, Female, Fetal	5.7	Skeletal Muscle, Male, Adult	0.2
Kidney, Female, Adult	3.1	Skeletal Muscle, Male, Fetal	2.4
Skin, Female, Adult	0.92	Testes, Male, Adult	8.6
Skin, Female, Fetal	2.6	Thymus, Male and Female, Fetal	1.6
Skin, Male, Adult	1	Thymus, Male, Adult	1.7
Spleen, Female, Adult	1.8	Thyroid, Female, Adult	1.3
Spleen, Female/Male pooled, Fetal	7.7	Tongue, Male/Female, Adult	1.9
Spleen, Male, Adult	2.4	**Trachea, Female, Adult**	**5.5**
Stomach, Female, Adult	0.18	**Uterus, Female, Adult**	**13.7**
Stomach, Female, Fetal	6.2	Colon, Female, Adult (Top)	3
Stomach, Male, Adult	0.6	Larynx, Male, Adult (Normal)	5.5
Stomach, Male, Fetal	3.9		

### Cytokines increase oxytocin receptor expression in HASMCs

**Figure 2:**
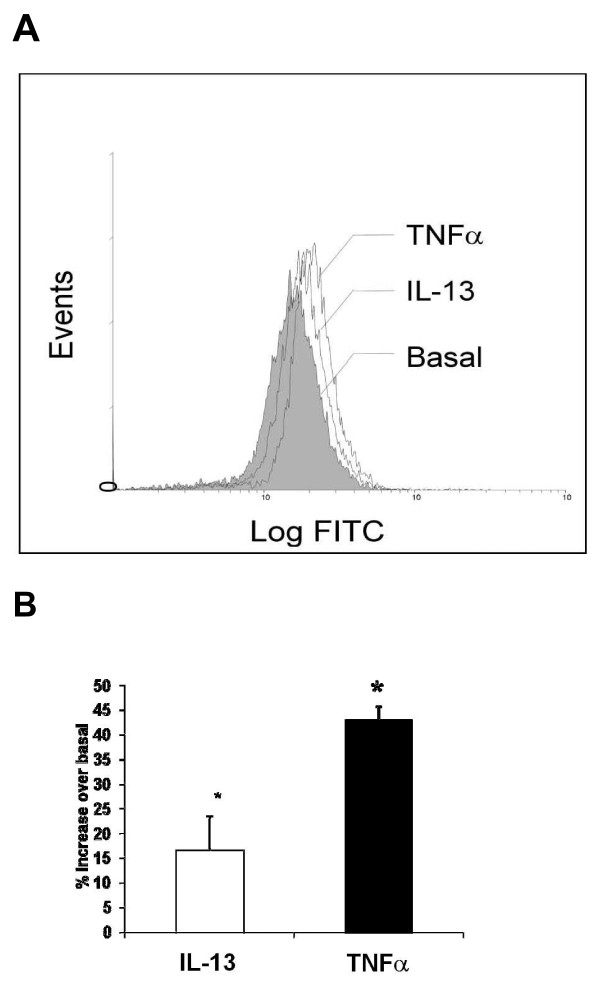
**(A) Representative flow-cytometric analysis of oxytocin receptor expression in basal and HASM cells exposed to 50 ng/ml IL-13 or 10 ng/ml TNFα for 24 hr**. (B) Graphical representation of the data as percentage increase in mean fluorescence intensity over basal. *P < 0.05 as compared with untreated cells, n = 9.

In order to confirm the stimulatory effect of IL-13 and TNFα on oxytocin receptor at the protein level, flow cytometry was performed. As shown in Figure 2, expression of oxytocin receptor was significantly augmented in cells treated with either TNFα (10 ng/ml) or IL-13 (50 ng/ml) for 24 hr with a net 43 ± 2.8% and 16.7 ± 6.8% increase over basal, respectively (P < 0.05, n = 9). Increases in oxytocin receptor proteins by TNFα or IL-13 correlated with changes in total mRNA levels (Figures 1A and 1B), suggesting that inflammatory cytokines possibly upregulate oxytocin receptor in HASMCs at the transcriptional level.

### Inflammatory cytokines modulate oxytocin-evoked calcium mobilization

**Figure 3:**
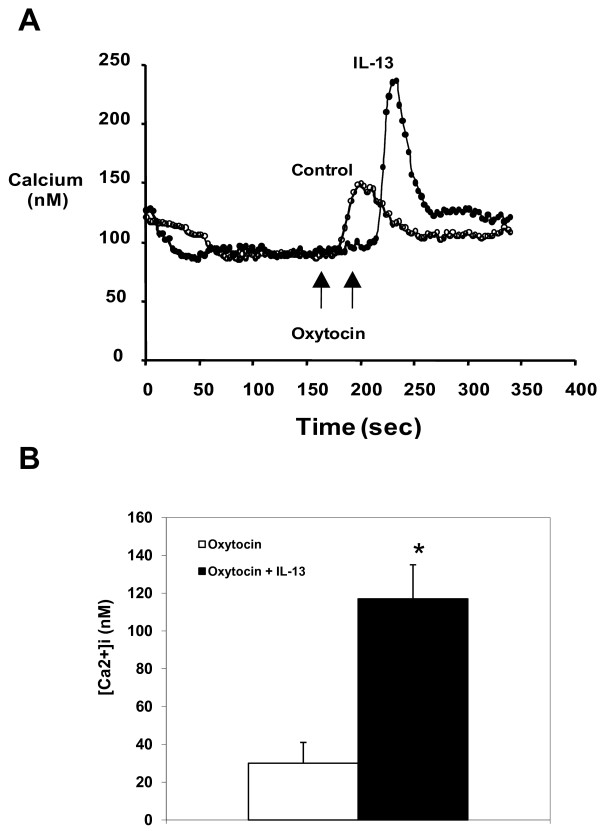
**Effect of IL-13 on oxytocin-evoked cytosolic free Ca^2+ ^concentration ([Ca^2+^]_i_)**. IL-13 (50 ng/ml) was pre-incubated for 24 hr before cells were exposed to 100 nM oxytocin. (A) Typical Ca^2+ ^traces from cells incubated in the absence or presence of IL- 13. (B) Graphical representation of values for the Ca^2+ ^peak phase from cells incubated in the absence or presence of IL-13. Results are expressed as the net increase in [Ca^2+^]_i _over basal (unstimulated) levels. Values are means ± SE of 3 separate experiments and are significantly different from oxytocin only control (P < 0.01). TNFα did not have a modulatory effect on oxytocin-induced calcium response in these cells.

**Figure 4:**
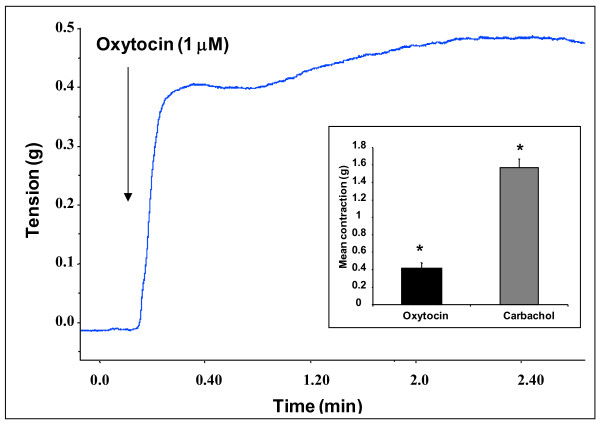
**Representative trace showing the contractile response induced by oxytocin in isolated murine tracheal rings**. Similar responses were observed in eight different tracheal rings. The insert shows the contractile responses expressed as means ± SEM from 8 different tracheal rings stimulated with 1 μM oxytocin or 10 μM carbachol (p < 0.05 compared to basal values, statistical significance using ANOVA).

We next determined whether oxytocin receptors expressed on the surface of HASM cells were functional by assessing oxytocin effects on mobilization of intracellular calcium and whether oxytocin calcium responses were modulated by cytokines. As shown in Figure. 3A and 3B, oxytocin (100 nM) induced a rapid increase in intracellular calcium concentration reaching 30 ± 11 nM that was significantly enhanced in IL-13-treated cells to 117 ± 18 nM (n = 9, P < 0.05) (Figure 4). Interestingly, although TNFα was more effective in increasing oxytocin receptor expression, oxytocin had no modulating effect on oxytocin-induced calcium responses in HASM cells. In contrast, TNFα significantly increased calcium signals in response to bradykinin from 454 ± 24 nM to 660 ± 59 nM (n = 3 different cell lines, P < 0.05).

### Oxytocin induces force generation and airway narrowing in murine tracheal ring and lung slices, respectively

We have previously shown that isolated murine tracheal rings represent an interesting *ex vivo *model to investigate the factors and the mechanisms that modulate airway smooth muscle responsiveness [32-34]. As shown in Figure 4, oxytocin elicited a rapid contractile response in murine tracheal rings (0.4 ± 0.06 g, n = 8, P < 0.05). The contractile response induced by oxytocin was sustained for 30 min (data not shown) but was much less robust as compared with that induced by carbachol, reaching 25% when expressed as an percentage (%) of the responses induced by 10^-5 ^M carbachol (1.57 ± 0.1 g). These data show that oxytocin is an effective contractile agonist in the airways.

**Figure 5:**
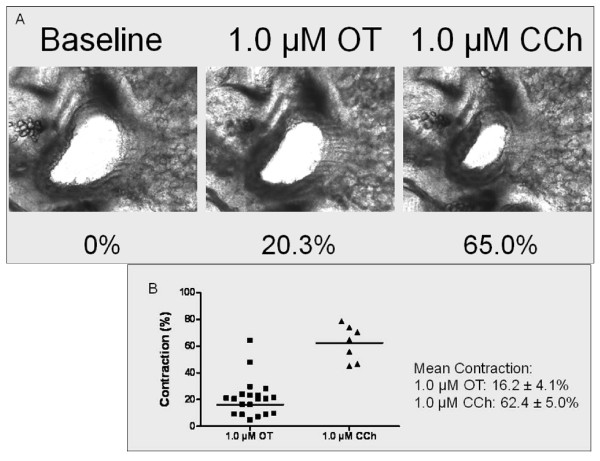
**Effect of oxytocin (OT) on murine intra-pulmonary airways with respect to a sub-maximal dose of carbachol (CCh)**. (A) Representational images show a murine airway at baseline (0% contraction), following 1.0 μM OT (20.3%) and 1.0 μM CCh (65.0%). (B) Maximum contraction of individual airways following OT and CCh. Mean ± SEM values of the data shown.

We also found that murine intra-pulmonary airways narrowed 16.2 ± 4.1% from baseline after the administration of 1.0 μM oxytocin. In separate experiments, the effects of oxytocin on carbachol-mediated force generation were examined. The airway was contracted maximally to oxytocin, and then carbachol was added. Carbachol further narrowed the airway to 62.4 ± 5.0% from baseline. Thus, the oxytocin contraction was 26% as effective as carbachol but does suggest that oxytocin has a contractile effect on airway smooth muscle in murine intra-pulmonary airways (Figure 5).

### Oxytocin levels in BAL samples from asthmatic and healthy subjects

The goal of these experiments was to assess whether oxytocin levels are determined and detected in the BAL of healthy subjects and those with asthma. As shown in Table 2, oxytocin levels are present in BAL fluid from both normal subjects and those with asthma (n = 10 in each group) but no significant changes were detected between these cohorts. Further, there was no gender differences in oxytocin levels observed. Oxytocin levels in sputum supernatant did not differ between asthmatics and healthy control subjects (median oxytocin levels 13.2 mcg/ml and 12.4 mcg/ml, respectively, p = 0.97). Multivariable analyses confirmed that there was no significant difference in oxytocin levels even after controlling for age, sex, percent predicted forced expiratory volume in one second, and inhaled corticosteroid dose.

**Table 2:** Oxytocin levels in BAL samples from asthmatic and healthy subjects

Asthmatics	Age	FEV1%pred	FEV1/FVC	Bronchodilator response (% change from baseline)	PC20 (mg/dl)	Gender	GINA	Inhaled Corticosteroid BDP equivalent	Oxytocin
1	31	120	82	3	0.1	M	1	**0**	14.0

2	47	96	73	16	1.6	F	1	**0**	12.9

3	40	69	60	15	0.7	M	1	**0**	12.4

4	43	86	80	8	1.6	M	1	**0**	13.4

5	52	72	68	12	2.4	F	1	**0**	21.3

6	28	90	85	6	0.6	F	1	**0**	15.0

7	31	80	72	15	3.2	M	1	**0**	10.1

8	41	68	66	18	1.4	F	1	**0**	10.9

9	36	86	83	6	0.2	F	2	**400**	19.5

10	68	97	60	15	0.5	F	2	**400**	9.5

**Mean ± SD**	**41.7 ± 11.9**	**86.4 ± 15.8**	**72.9 ± 9.4**	**11.4 ± 5.2**	**1.23 ± 1.0**				**13.9 ± 3.9**

**Controls**									

1	26	96	83	0	32	M	**0**	11.7	

2	30	112	88	0	32	F	**0**	12.4	

3	43	122	82	0	32	F	**0**	15.6	

4	55	100	78	1.7	32	F	**0**	12.3	

5	61	97	71	-4.8	32	F	**0**	11.4	

6	43	125	85	0	32	M	**0**	17.4	

7	47	108	85	0	32	M	**0**	12.3	

8	49	102	88	0	32	M	**0**	15.6	

9	45	106	78	4.3	32	F	**0**	17.1	

10	23	94	80	3.2	32	F	**0**	12.2	

**Mean ± SD**	**42.2 ± 12.4**	**106.2 ± 10.7**	**81.8 ± 5.2**	**0.44 ± 2.4**	**32 ± 0**				**13.8 ± 2.3**

## Discussion

Oxytocin regulates gonadal function such as labor-associated uterine contractions and milk discharge during lactation [1], but the role of oxytocin in other tissues remains unexplored. Evidence shows that the incidence of asthma switches at puberty from male to female predominance, and in adult asthma the ratio is about 2:1 in favor of women [38,39] and that asthma worsens in about 30% of pregnant women [9]. Whether oxytocin plays a role in determining gender susceptibility to asthma remains unknown. Our present study demonstrates the existence of functional oxytocin receptors on HASMCs and their regulation by pro-inflammatory cytokines known to be involved in asthma pathogenesis. Further, we report that oxytocin induced force generation and contractile responses in isolated murine tracheal rings [32-34] and lung slices [40,41]. These data suggest that expression of oxytocin receptor (OXTR) on HASMCs can be differentially modulated by inflammatory cytokines in HASMCs, a mechanism that may contribute to the altered airway responsiveness observed in asthma assuming that such receptor changes would occur in vivo.

The study of the molecular mechanisms regulating the expression of oxytocin receptor remains complex [42,43]. Most studies that attempt to elucidate the transcriptional regulation of oxytocin receptors have been performed in myometrial and uterine cells. Using these models, investigators found that OXTR expression was downregulated by IL-1β, IL-6 but not by TNFα [44,45]. Others found that lysophospholipids increased translation of oxytocin receptor possibly as a consequence of increased mRNA stability [46]. Our report is the first to demonstrate that expression of oxytocin receptor is increased in HASMCs treated with pro-inflammatory cytokines, either TNFα or IL-13. An increased gene transcription was detected by the real time PCR data demonstrating a rapid effect of these cytokines on oxytocin receptor expression. In addition, both TNFα and IL-13 up-regulated receptor expression at the protein level with a 7 fold and 3.5 fold increase over basal, respectively. Unexpectedly, IL-13, but not TNFα, enhanced oxytocin-evoked calcium responses in HASMCs. The mechanisms responsible for the differential effects of TNFα and IL13 to enhance oxytocin-evoked calcium responses remain unclear. This is surprising given evidence that IL-13 and TNFα comparably enhance calcium signals induced by bradykinin and acetylcholine [19,34,47,48]. Our data does support the hypothesis that TNF and IL-13 have disparate effects on OXTR expression and that agonists with less efficacy at inducing calcium mobilization may be enhanced by increases in receptor expression rather than by processes that amplify downstream pro-contractile signaling pathways. Accordingly, we and others also reported that changes in bradykinin receptor expression in part explained the enhancing effects of cytokines (TNFα or IL-1β) on agonist-evoked calcium responses [49,50]. In some but not all instances, modulation of receptor expression may not correlate with receptor function in ASM cells. In previous reports, we and others found that although calcium signals to acetycholine were significantly increased by TNFα, muscarinic receptor density were significantly reduced in TNFα-treated cells [51,52]. Collectively, these data suggest that cytokine-induced changes in receptor function may be uncoupled from cell surface receptor density. Additionally, signaling pathways such as alterations in calcium sensitization could also contribute to the observed differences in cytokine-induced agonist responsiveness but such mechanisms require further study.

We show that oxytocin receptors are expressed on HASMCs and cytokines modulate receptor expression [48]. In myometrium cells, TNFα enhanced oxytocin-induced calcium transients [20]. Whether TNF effects were due to alterations in receptor expression was not addressed. These investigators, however, found that CD38 played a critical role in the enhanced oxytocin-induced calcium responses in myometrium cells. Interestingly, both TNFα and IL-13 also upregulated CD38 expression and function in HASMCs [48,53-56]. Because IL-13, but not TNFα, enhanced oxytocin calcium responses in HASMCs, our findings possibly suggest that both CD38-dependent and independent pathways may contribute to cytokine-induced alterations in oxytocin responses.

Using two *ex vivo *models to study airway responsiveness, namely, isolated tracheal rings [35] and mouse lung slices [40,41], we found that oxytocin induced ASM contraction and airway narrowing, suggesting that oxytocin serves as a bronchoconstrictor. Whether these responses, which are modest in magnitude compared to those induced by carbachol, are clinically relevant remains unknown. Our study, however, shows that these bronchoconstrictor responses induced by oxytocin were dramatically increased by IL13. However, we failed to detect any effects of IL-13 on oxytocin-evoked contractile responses (Amrani et al., unpublished observations). The reasons explaining the discordance observed between IL-13 effects on cultured cells and on ex vivo tissue remains unexplained but could reflect that IL-13 effects in complex tissue provides negative homeostatic effects that dampens IL13's ability to enhance oxytocin-induced bronchoconstriction. Alternatively, the diffusing barrier of the tissue thickness mitigates the ability of IL-13 and/or oxytocin to stimulate their cognate receptors. Accordingly, contractile responses to oxytocin could be modulated by effects on airway epithelial cells and/or vascular endothelium [16] or by subsequent production of relevant factors such as nitric oxide [43]. Further studies are needed to address whether IL-13 modulates oxytocin effects in other lung cells.

Given our observations that oxytocin induced airway smooth muscle contraction and that cytokines increased expression of the receptor, we characterized whether BAL fluid derived from healthy subjects as well as subjects with asthma had detectable oxytocin levels. Our studies revealed that detectable oxytocin levels were found in the BAL fluid in both cohorts; however, in stable asthma, there was no increase in oxytocin levels in BAL fluid. We postulate that the mechanism by which oxytocin may play a role in asthma in acute exacerbations concerns enhanced vascular permeability into tissue where the receptor number in ASM but not the ligand are markedly increased. To address this hypothesis, OXTR expression in ASM tissue derived from subjects with acute exacerbation would be required but such studies are beyond the scope of the current study.

In summary, our report provides the first evidence of a contractile role of oxytocin in the airways. Future studies will address the nature of the common transcription factors as well as signaling pathways by which both cytokines regulate the transcription of the oxytocin receptor gene. Since gender disparity in asthma is well recognized, studies could also address whether differential OXTR expression in the airways between men and women in part explains gender differences in asthma prevalence and morbidity or whether differential OXTR expression mediates asthma morbidity in pregnancy.

## Competing interests

The authors declare that they have no competing interests.

## Authors' contributions

FS, CH, KI, VL performed the PCR assays, DJ, SK, HB did the calcium experiments, contractility studies and flow cytometry assays, respectively. PRP carried out the PCLS. HZ performed the ELISA in the BAL samples. SS and CB provided the BAL samples. YA, FS, DG, LL, RAP participated in the design and coordination. YA, FS, RAP conceived the study and wrote the manuscript. All authors read and approved the final masniscript.
